# Psychoactive Pollutant Impacts Baseline Immune Traits but Not Behavior and Microbiome in a Fish

**DOI:** 10.1021/acs.est.6c12057

**Published:** 2026-10-20

**Authors:** Gabriel Melhado, Rhiannon V. Eastment, Francine Z. Marques, Upama Aich, Anne Peters, Bob B. M. Wong

**Affiliations:** † School of Biological Sciences, 2541 Monash University, Melbourne, Victoria 3800, Australia; ‡ School of Medical Sciences, Faculty of Biology, Medicine, and Health, University of Manchester, Manchester, Greater Manchester M13 9PL, United Kingdom; § Department of Pharmacology, Biomedicine Discovery Institute, Faculty of Medicine, Nursing and Health Sciences, Monash University, Melbourne, Victoria 3800, Australia; ∥ Victorian Heart Institute, Monash University, Clayton, Victoria 3168, Australia; ⊥ Baker Heart and Diabetes Institute, Melbourne, Victoria 3000, Australia; # Centre for Evolutionary Biology, School of Biological Sciences, The University of Western Australia, Perth, Western Australia 6009, Australia

**Keywords:** aquatic pollution, anthropogenic contaminants, fluoxetine, ecoimmunology, behavioral ecology, fish physiology, microbiome

## Abstract

Pharmaceutical pollution is an increasing global threat to freshwater ecosystems. Psychoactive drugs, such as antidepressants, are of particular concern because they affect evolutionarily conserved neuroendocrine pathways in nontarget organisms. As these pathways regulate interconnected biological systems, understanding the ecological consequences of pharmaceutical exposure requires an integrative approach. Here, we investigated the effects of 28-day exposure to control (freshwater), low (4.2 ± 0.3 ng/L), and high (637.5 ± 173.84 ng/L) fluoxetine concentrations on behaviors, baseline immune traits, and skin mucus microbiome composition in the Murray River rainbowfish (*Melanotaenia fluviatilis*). Fluoxetine exposure did not impact fish behavior, including activity, freezing behavior, shelter use, or microbiome composition. In contrast, immune traits were significantly altered. Both fluoxetine concentrations reduced total white blood cell (WBC) count, while only fish exposed to the low concentration showed reduced lymphocyte counts and elevated neutrophil counts, consistent with a stress-associated WBC redistribution response. Our findings show that different, but interconnected, biological systems can differ in their sensitivity to pollutants and suggest that immune traits may be an early, sensitive indicator of exposure to psychoactive pharmaceuticals. These findings highlight the importance of integrating multiple biological end points to obtain a more comprehensive understanding of the ecological consequences of pharmaceutical contamination.

## Graphical abstract

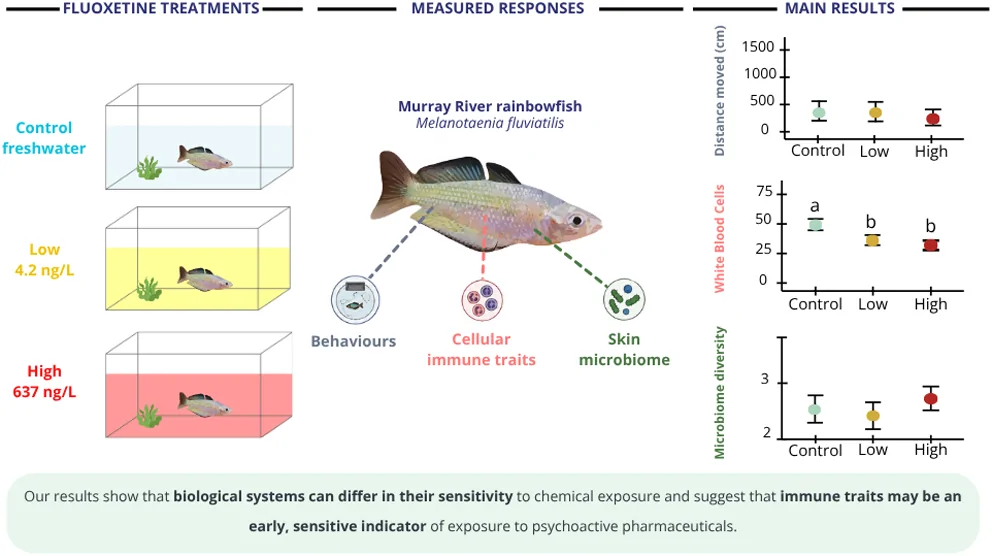

## 1 Introduction

Pharmaceutical pollution is increasingly pervasive in freshwater ecosystems worldwide, raising concerns about its ecological impacts on aquatic wildlife.^1–4^ Many pharmaceuticals can enter aquatic environments due to incomplete removal during wastewater treatment.^5,6^ Among these contaminants, psychoactive pharmaceuticals are especially concerning because they target neuroendocrine pathways that are evolutionarily conserved across vertebrates, potentially leading to unintended effects on the behavior and physiology of nontarget organisms.^5,7^ Fluoxetine, commonly sold under the brand name Prozac, is a particularly relevant psychoactive pharmaceutical because it is one of the world’s most widely prescribed antidepressants and is frequently detected in freshwater environments.^5,6,8^ As a selective serotonin reuptake inhibitor (SSRI), fluoxetine increases extracellular serotonin levels and can thus alter behavioral and physiological regulation.^5,6^ For instance, fluoxetine has been shown to impact antipredator behavior, anxiety-related responses, and circadian activity in fishes.^9–11^ It has also been reported to induce oxidative stress, reduce hormone production, and alter phagocytic immune responses across aquatic taxa.^12–15^ In rats, fluoxetine can also alter the proportions of different types of lymphocytes, including CD4+ and CD8+ T cells, and the chemical signals within these cells.^16^ In human cell cultures, fluoxetine can affect signaling between immune cells, specifically macrophages and CD4+ T cells, through mechanisms involving serotonin receptors.^17^ Although fluoxetine can affect both behavioral and physiological responses, these responses may not be equally sensitive to exposure despite being mediated by shared neuroendocrine mechanisms.^18–21^ For example, chronic exposure to fluoxetine altered swimming behavior in zebrafish (*Danio rerio*) without affecting neurotransmission or metabolism.^20^ This highlights the importance of integrating behavioral and physiological end points to obtain a more comprehensive understanding of how psychoactive pollutants affect aquatic organisms.

Changes in behavior and physiology can have successive effects on other biological systems through feedback loops involving immune regulation and stress physiology.^22,23^ In aquatic organisms, these interactions can extend to host-associated microbial communities, particularly those inhabiting mucosal surfaces, such as the skin mucus, which functions as a first barrier against pathogens and other environmental stressors.^24,25^ Host immune regulation can shape microbial composition and stability through changes in barrier function, homeostasis, and stress physiology, while microbial communities can, in turn, influence immune activity, metabolism, endocrine signaling, and behavioral responses through bidirectional microbiome–host interactions.^26–29^ Although these relationships are well established for the gut microbiome, considerably less is known about whether similar interactions occur within the skin microbiome of aquatic vertebrates. Importantly, environmental stressors, such as pharmaceutical pollution, can affect these feedback loops by simultaneously altering host physiology, behavior, and microbial community structure.^30,31^ In fishes, fluoxetine has been shown to alter gut microbial community composition at relatively high experimental concentrations,^32^ yet its effects on the skin microbiome remain largely unexplored. Consequently, fluoxetine-induced physiological changes may have cascading effects across multiple levels of biological organization, ultimately affecting pathogen susceptibility, disease dynamics, fitness, and survival.

Despite growing evidence that fluoxetine can influence multiple biological systems in wildlife, few studies have simultaneously investigated behavioral, physiological, and microbial responses within a single experimental framework, thereby limiting our understanding of how these interconnected systems collectively shape organismal responses to pharmaceutical exposure. Here, we investigated the effects of fluoxetine exposure on locomotor activity, anxiety-related and risk-taking behaviors, baseline cellular immune traits, and skin mucus microbiome composition in the Murray River rainbowfish (*Melanotaenia fluviatilis*), an emerging model species in aquatic ecotoxicology.^33–36^ Based on previous studies showing that fluoxetine impacts activity, antipredator responses, and anxiety-related behaviors in other fish species,^9–11,20^ we predict that exposure of rainbowfish to fluoxetine will reduce locomotor activity, increase risk-taking behavior, and alter stress-associated behavioral responses. Importantly, because the same pathways that underpin behavioral changes also regulate immune function, we hypothesize that exposure to fluoxetine would alter circulating white blood cell (WBC) profiles, which are widely used as a measure of immune status in vertebrates.^37^ In addition, we hypothesize that fluoxetine exposure would alter the diversity and composition of skin mucus microbial communities, as previous evidence suggests that fluoxetine can modify host-associated microbial communities and disrupt microbiome homeostasis.^32^ By integrating behavioral, physiological, and microbial end points, our study aims to contribute to a more comprehensive understanding of how pharmaceutical pollutants affect interconnected systems that collectively influence organismal health, disease susceptibility, and ecological performance.

## 2 Materials and Methods

### 2.1 Housing and Exposure Protocol

#### 2.1.1 Fish Husbandry

Unsexed juvenile rainbowfish (*M. fluviatilis*; *N* = 160) used in this study were purchased commercially (Middle Creek Farm, Victoria, Australia) and acclimated to laboratory conditions (23–25 °C; 12:12 h light/dark cycle) for 3 weeks before experimentation in glass aquaria (60 × 30 × 30 cm^3^, 54 L) containing carbon-filtered freshwater. During housing, fish were fed *ad libitum* once daily with commercial fish food (FKC, Queensland, Australia) and newly hatched live brine shrimp *Artemia sp.*.^38^ The present research was approved by the Biological Sciences Animal Ethics Committee of Monash University (2024–39278–106106).

#### 2.1.2 Exposure Protocol

After acclimation, fish were exposed to fluoxetine for 28 days in a closed-system setup with 24 separate tanks, following protocols adapted from Martin et al.^10^ Fish were randomly allocated to one of three treatments: (1) a freshwater control without fluoxetine (*N* = 54), (2) a low fluoxetine concentration (nominal: 400 ng/L; *N* = 53), or (3) a high fluoxetine concentration (nominal: 4000 ng/L; *N* = 53). Each treatment had 8 replicate tanks, each containing 6–7 fish. Tanks were maintained as independent experimental units, each with its own filtration and aeration system, with no water exchange between tanks or treatments. Lighting and feeding conditions were the same as those described for acclimation. The selected concentrations of fluoxetine reflect environmental levels in freshwater, with the low concentration representing levels downstream of wastewater discharges and the high concentration representing levels detected in treatment plant effluent, capturing a realistic exposure gradient.^39,40^ The 28-day exposure period was selected to capture chronic effects of fluoxetine, consistent with the temporal dynamics of SSRIs, which typically require 2–4 weeks to induce physiological and behavioral responses in humans.^41,42^

#### 2.1.3 Stock Solution Preparation and Chemical Analysis

Stock solutions were prepared following protocols adapted from Bertram et al.^43^ to achieve low- and high-fluoxetine treatment concentrations. Fluoxetine hydrochloride (CAS: 56296–78–7; Sigma-Aldrich, St. Louis, MO) was dissolved in methanol (HPLC-grade, ≥99.9%) and diluted with Milli-Q water to prepare a concentrated stock solution (15 mg/L). Working stock solutions for each treatment were subsequently prepared by dilution to achieve the desired nominal exposure concentrations. The exposure concentrations in the housing tanks were maintained through partial water changes, in which 30% of the tank volume was replaced twice weekly with aged and conditioned water. After each water change, the volume of fluoxetine stock solution added to each tank was calculated based on the tank’s remaining water volume to maintain exposure concentration. To verify exposure levels, water samples were collected from all treatment tanks twice weekly throughout the experimental period. Samples were analyzed by Envirolab Services (Perth, Australia) using gas chromatography coupled to tandem mass spectrometry (GC–MS/MS), following established analytical methods.^44^

### 2.2 Behavioral Trials

After exposure, fish were transferred individually from their housing tanks to a behavioral arena consisting of a glass tank (30 × 15 × 15 cm^3^) containing a shelter zone created by a covered area above the water surface (15 × 5 cm^2^) (Figure 1A). Behavioral trials were conducted using aged and conditioned freshwater, and water depth was standardized across all trials (4 cm). Fish were first placed into a confined container within the arena and allowed to acclimate for 5 min. Following acclimation, the container was removed, allowing individuals to explore the arena freely. Behavior was recorded from above for 20 min using a digital video camera (Panasonic HC-V180). To minimize the accumulation of chemical and social cues, 50% of the arena water was replaced between consecutive trials using fresh, aged and conditioned water. Video recordings were analyzed using EthoVision XT 12.0 (Noldus Information Technology, The Netherlands). We measured three behavioral traits commonly used to assess activity, stress-related responses, and risk-taking behavior in fish because they capture behavioral responses that are frequently affected by psychoactive contaminants and other environmental stressors:^45,46^ (i) total distance traveled (cm), used as a measure of locomotor activity; (ii) time spent freezing (s), defined as the absence of movement in the open arena and commonly used as an indicator of anxiety-related or stress-associated behavior; and (iii) time spent in the shelter (s), which represents refuge use and risk-avoidance behavior.

**Figure 1:**
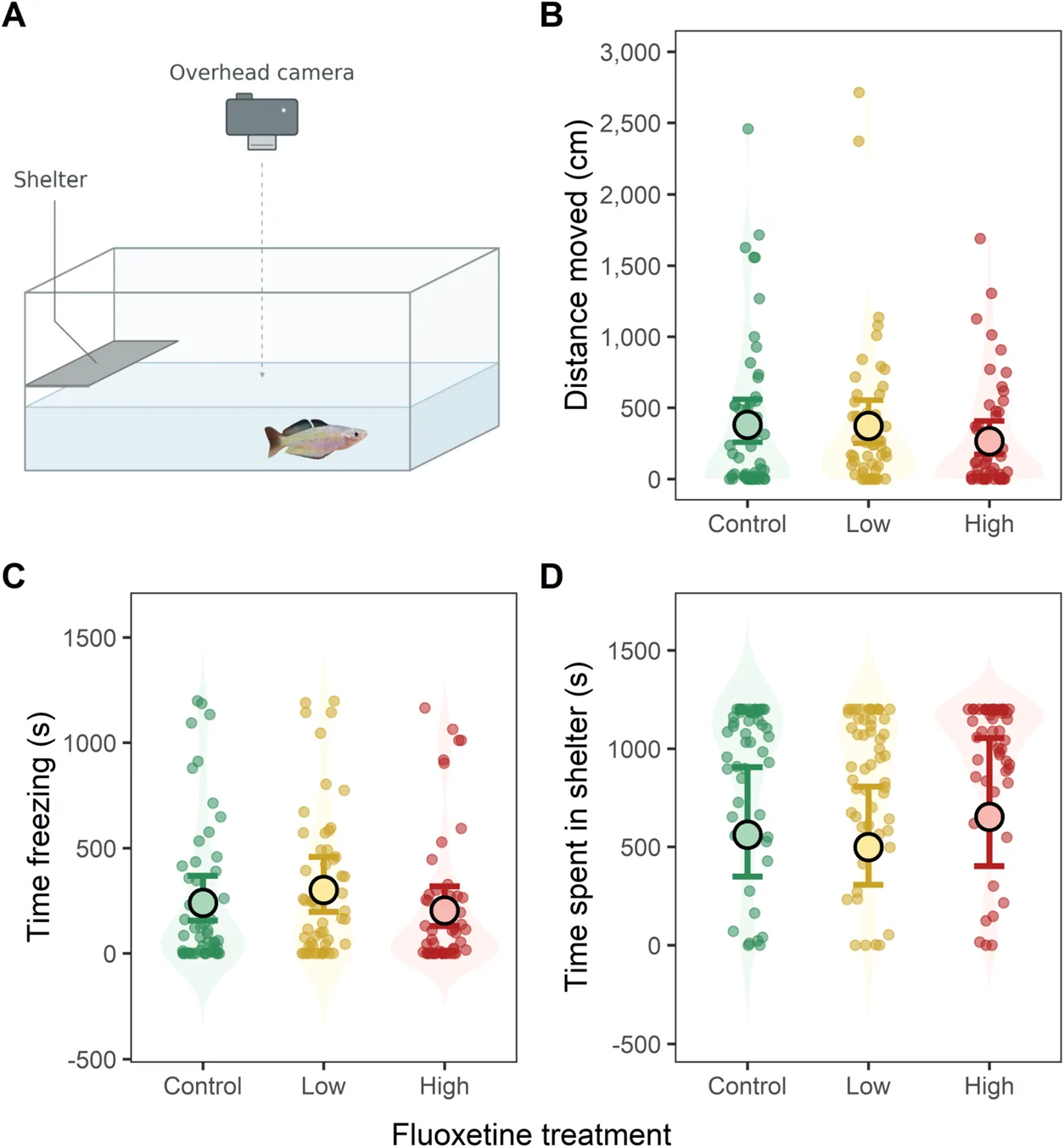
*Fluoxetine exposure does not significantly affect behavioral responses*. (A) Schematic of the behavioral arena. Violin plots representing (B) distance moved (cm), (C) time spent freezing (s), and (D) time spent in shelter (s) following 28 days of exposure to control, low, and high fluoxetine concentrations. Colored points indicate individual fish, and large circles with error bars represent estimated marginal means ±95% confidence intervals derived from generalized linear mixed-effects models. *N* = 160 fish (Control = 54, Low = 53, High = 53).

### 2.3 Skin Mucus Microbiome

To assess whether fluoxetine exposure altered the diversity and structure of host-associated microbial communities, we collected skin mucus samples from rainbowfish following established protocols.^47^ Specifically, after the behavioral trials, fish were euthanised with an overdose of Aqui-S (40 mg/L), and mucus was sampled from the lateral surface of each individual using sterile swabs and immediately stored at −80 °C for downstream molecular analyses. To monitor potential contamination associated with low-biomass mucus samples, negative controls were included during sample processing. These consisted of extraction blanks containing reagents only, and sterile swab controls processed without contact with fish mucus. Microbial DNA was extracted using the DNeasy PowerSoil Kit (Qiagen, Germany) following the manufacturer’s protocol with minor modifications, in which mucus volume was used in place of the standard soil input. Bacterial community composition was then characterized using full-length 16S rRNA gene sequencing. Libraries were prepared and sequenced using PacBio HiFi technology at the Australian Genome Research Facility, enabling high-resolution taxonomic assignment. Raw sequence data were processed in QIIME2,^48^ with sequence denoising and amplicon sequence variant (ASV) inference performed using DADA2.^49^ Taxonomic classification was conducted using a naïve Bayes classifier trained on the GTDB r207 and SILVA v138 reference databases for bacterial assignment. Sequences identified as nonbacterial (e.g., chloroplasts and mitochondria) were removed prior to downstream analyses. ASVs present in fewer than 10% of samples were additionally filtered to reduce sparsity and minimize the influence of rare taxa. Samples with fewer than 1000 reads following quality filtering were excluded before downstream analyses. Following the filtering steps, 121 out of 129 samples and 261 ASVs were retained for analysis, representing 79.6% of total reads and a mean sequencing depth of 24,191 reads per sample (range = 1050–45,727).

Microbiome community analyses were conducted using the phyloseq framework.^50^ Separate data objects were generated for diversity analyses,^51^ in which ASV counts were transformed to relative abundances using total sum scaling (TSS). For compositional analyses, count data were additionally transformed using a centered log-ratio (CLR) transformation to account for the compositional nature of microbiome data and differences in sequencing depth among samples.^51,52^

We quantified α diversity using observed richness (taxonomic richness), Shannon diversity (richness and evenness),^53,54^ and Simpson diversity (dominance and evenness)^55^ indices. β diversity was estimated using Euclidean distances calculated from CLR-transformed ASV data, which are equivalent to Aitchison distances for compositional microbiome data sets and quantify differences in microbial community composition among samples.^52^ Genus-level differential abundance was assessed using MaAsLin2 ^56^ to identify bacterial genera whose relative abundances differed among fluoxetine treatments.

### 2.4 Cellular Immune Traits

We assessed baseline cellular immune traits using circulating white blood cell (WBC) profiles, as changes in leukocyte counts and profile are widely used indicators of immune status in vertebrates.^37,57,58^ Immediately after mucus collection, each fish was weighed (±0.0001 g) and its standard length measured using digital callipers (±0.01 mm) (see Supporting Information *-* Table S1). Subsequently, a blood sample (∼10 μL) was collected from each fish via cardiac venipuncture. Blood smears were prepared and air-dried for 30 min before fixation. Slides were then fixed in methanol for 3 min and stained with Wright’s/Giemsa solution (WG80; Sigma-Aldrich) for 20 min. Following staining, slides were rinsed with distilled water and allowed to air-dry overnight before microscopic examination. WBC were counted across 10 fields of view, each containing 100 erythrocytes, at ×1000 magnification using a Motic BA210 microscope (USA). In addition, a differential WBC count was performed by classifying 100 leukocytes per smear as lymphocytes, neutrophils, monocytes, or eosinophils to calculate the absolute counts for each leukocyte type. Because monocytes represented only 3.1% of all leukocytes counted and eosinophils were not observed, subsequent statistical analyses focused on total WBC count, lymphocyte count, neutrophil count, and the neutrophil-to-lymphocyte (N/L) ratio. All slides were analyzed by a single observer (GM), blind to treatment.

### 2.5 Statistical Analysis

All statistical analyses were conducted in R version 4.6.0 (R Core Team, 2026). Data were inspected for distributional assumptions and outliers, and where necessary, response variables were log- or square-root-transformed to improve normality and homoscedasticity. Statistical significance was assessed at α = 0.05.

#### 2.5.1 Behavioral and Immune Traits

To test for treatment effects on behavioral and immune traits, we fitted separate generalized linear mixed-effects models (GLMMs) using the *glmmTMB* package.^59^ For behavioral data, as some individuals never emerged from the shelter during the trials, we first tested whether fluoxetine exposure affected the probability of emergence using a binomial GLMM, thereby distinguishing treatment effects on the likelihood of initiating exploration from effects on subsequent behavioral responses. Subsequent behavioral and immune analyses were conducted using GLMMs fitted to the full data set. Error distributions were selected for each response variable based on the data distribution and model diagnostics. Gaussian models were used for approximately normally distributed variables following transformation where necessary (i.e., total distance traveled, WBC count, lymphocyte count, and neutrophil count), whereas Tweedie distributions were used for zero-inflated and right-skewed response variables (i.e., freezing time, shelter use, and neutrophil-to-lymphocyte ratio).^60^ Fluoxetine treatment (control, low, high) was included as a fixed effect in all models. Body mass was included as a continuous covariate to account for size-dependent variation. Tank was included as a random intercept to account for any potential tank effect. Model assumptions were evaluated using simulation-based residual diagnostics implemented in the *DHARMa* package.^61^ Post hoc pairwise comparisons among treatments were conducted using estimated marginal means with Tukey adjustments for multiple comparisons, implemented via the *emmeans* package.^62^

#### 2.5.2 Microbiome

Differences among treatments in α diversity indices were tested using GLMMs that included treatment as a fixed effect, sequencing depth (log-transformed read counts) as a covariate to account for differences in sequencing effort, and tank as a random intercept. Differences in community composition among treatments were tested using permutational multivariate analysis of variance (PERMANOVA) with 999 permutations, implemented in the vegan package.^63^ The differential abundance analysis included treatment and log-transformed sequencing depth as fixed effects and tank as a random intercept. Raw counts were normalized using total sum scaling (TSS) and log-transformed, and *p*-values were adjusted using the Benjamini–Hochberg false discovery rate.^64^ Associations with *q* < 0.05 were considered statistically significant.

## 3 Results

### 3.1 Exposure Conditions

The mean measured fluoxetine concentration was 4.2 ± 1.08 ng/L (±SD; *N* = 16) for the low treatment and 637.5 ± 173.8 ng/L (*N* = 16) for the high treatment. No fluoxetine contamination was detected in the control tanks (all samples were below the quantification limit of 2 ng/L, *N* = 8). The analytical validation of fluoxetine concentrations is provided in the Supporting Information (Figure S1 and Table S2). Water temperature averaged 23.95 ± 0.16 °C (±SD), and the pH range was 6.5–7.0. No mortality occurred in any treatment group during the exposure period.

### 3.2 Behavioral Responses

Fluoxetine exposure did not affect the probability of fish emerging from the shelter during the behavioral trial (χ^2^ = 0.25, *df* = 2, *p* = 0.88). Consistent with this, fluoxetine exposure did not have a significant effect on distance moved (χ^2^ = 1.88, *df* = 2, *p* = 0.39; Figure 1B), freezing (χ^2^ = 1.66, *df* = 2, *p* = 0.44; Figure 1C), and shelter use behavior (χ^2^ = 2.11, *df* = 2, *p* = 0.35; Figure 1D).

### 3.3 Skin Mucus Microbiome

Fluoxetine exposure did not affect α diversity of the skin mucus microbiome across any of the indices measured (Figure 2). Observed richness did not differ among treatments (low: *z* = 0.67, *p* = 0.50; high: *z* = 0.95, *p* = 0.34). Similarly, neither Shannon diversity (*F*_2_,_17_._1_ = 0.12, *p* = 0.89) nor Simpson diversity (*F*_2_,_15_._6_ = 0.42, *p* = 0.67) varied among treatments. Sequencing depth did not affect Shannon diversity (*F*_1_,_112_._7_ = 1.28, *p* = 0.26) or Simpson diversity (*F*_1_,_114_._7_ = 1.69, *p* = 0.20).

**Figure 2:**
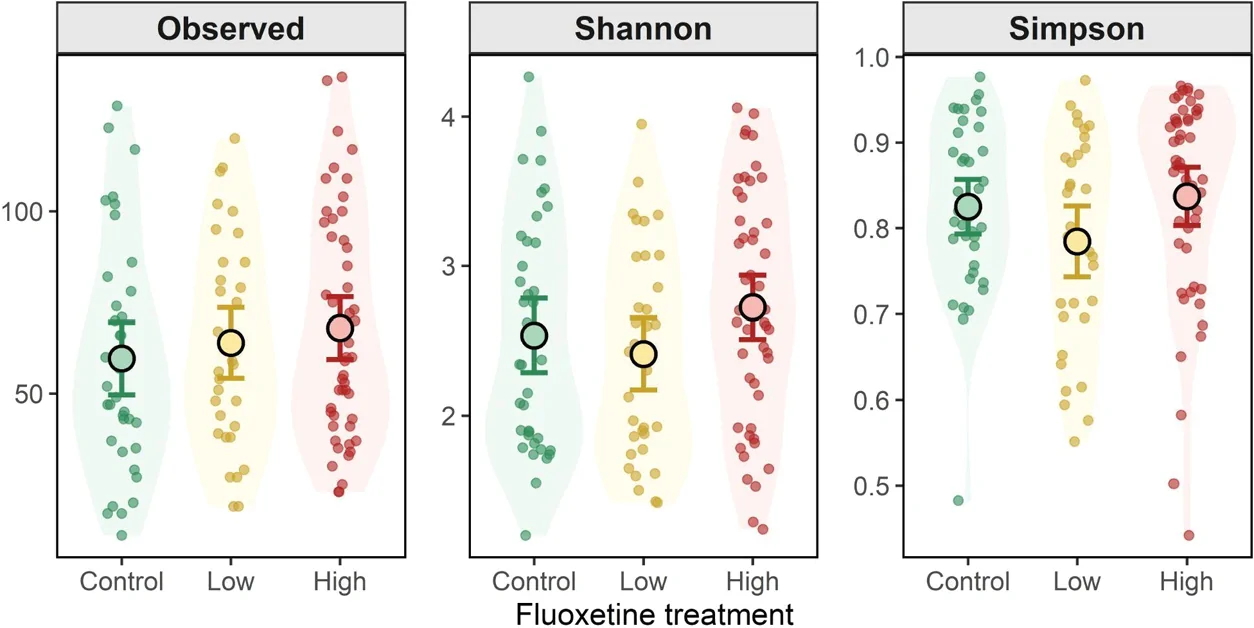
*Fluoxetine exposure did not alter α diversity of the skin mucus microbiome across three diversity indices.* Observed richness, Shannon diversity, and Simpson diversity for fish exposed to control, low, and high fluoxetine concentrations. Violin plots represent the distribution of individual observations, colored points indicate individual fish samples, and large circles with error bars represent mean values ±95% confidence intervals. Sample sizes after sequence quality filtering were *N* = 121 (Control = 38, Low = 33, and High = 50).

There was no evidence that microbial community composition differed among treatments. Analysis of Euclidean distances calculated from CLR-transformed ASV data revealed no significant effect of treatment on community composition (PERMANOVA, *R* ^*2*^ = 0.019, *F*_2_,_118_ = 1.14, *p* = 0.20) (Figure 3A). Similarly, genus-level differential abundance analysis detected no bacterial genera whose relative abundances differed significantly among treatments after false discovery rate correction (all *q* > 0.05). Proteobacteria was the dominant phylum across all treatments, followed by Firmicutes and Bacteroidota (Figure 3B).

**Figure 3:**
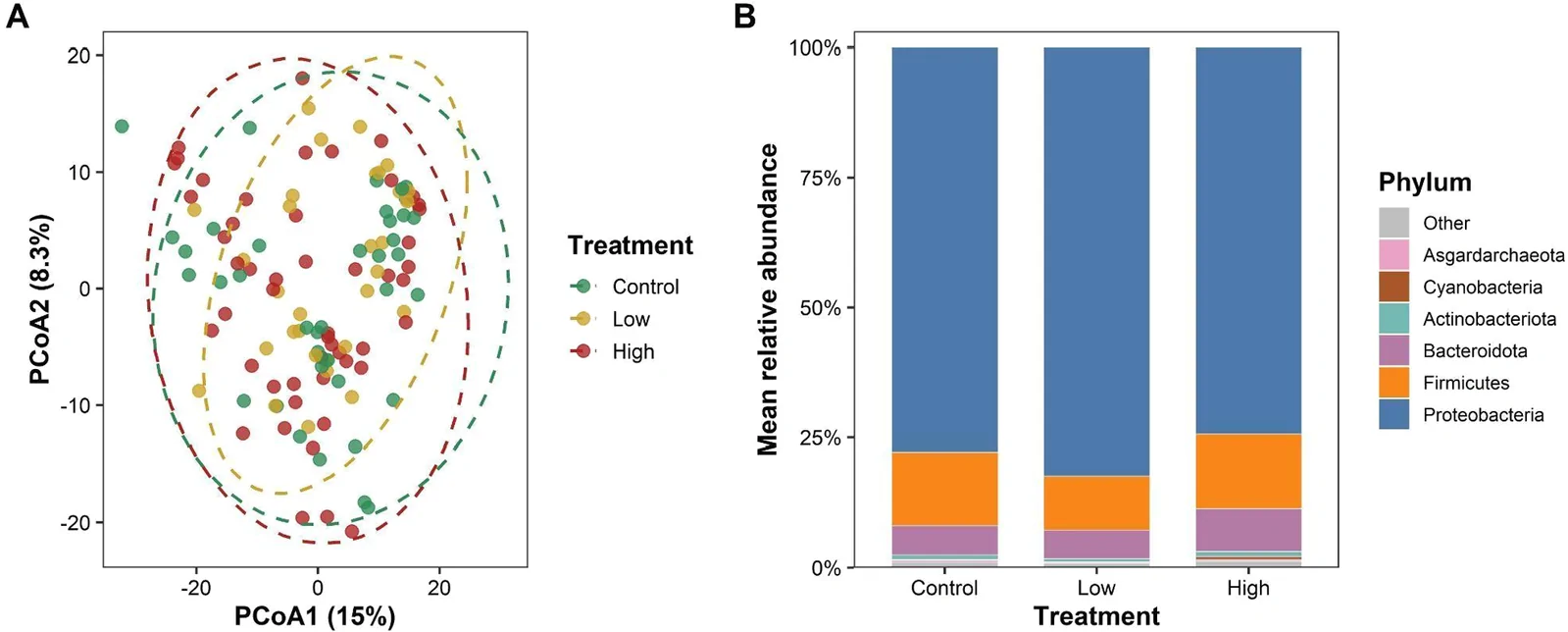
*Fluoxetine exposure did not alter the overall composition or dominant taxonomic structure of the skin mucus microbiome.* (A) Principal coordinates analysis (PCoA) based on Aitchison distances calculated from CLR-transformed β diversity showing substantial overlap in microbial community composition among control, low, and high fluoxetine treatments. Points represent individual fish samples, and dashed ellipses indicate treatment group dispersion (*N* = 121). Axis labels indicate the percentage of variation explained by each principal coordinate. (B) Mean relative abundance of dominant bacterial phyla across treatments, showing broadly similar taxonomic composition among groups. Colors represent different bacterial phyla, with low-abundance taxa grouped as “Other”. Sample sizes after sequence quality filtering were *N* = 121 (Control = 38, Low = 33, and High = 50).

### 3.4 Baseline Cellular Immune Traits

Fluoxetine significantly affected total white blood cell (WBC) count (χ^2^ = 32.64, *df* = 2, *p* < 0.001) (Figure 4A). Both fluoxetine-exposed groups had lower WBC count compared to the control (control vs low: estimate = 0.99 ± 0.25, *p* = 0.0003; control vs high: estimate = 1.36 ± 0.25, *p* < 0.0001). There was no significant difference between the low and high treatments (estimate = −0.37 ± 0.25, *p* = 0.30).

**Figure 4:**
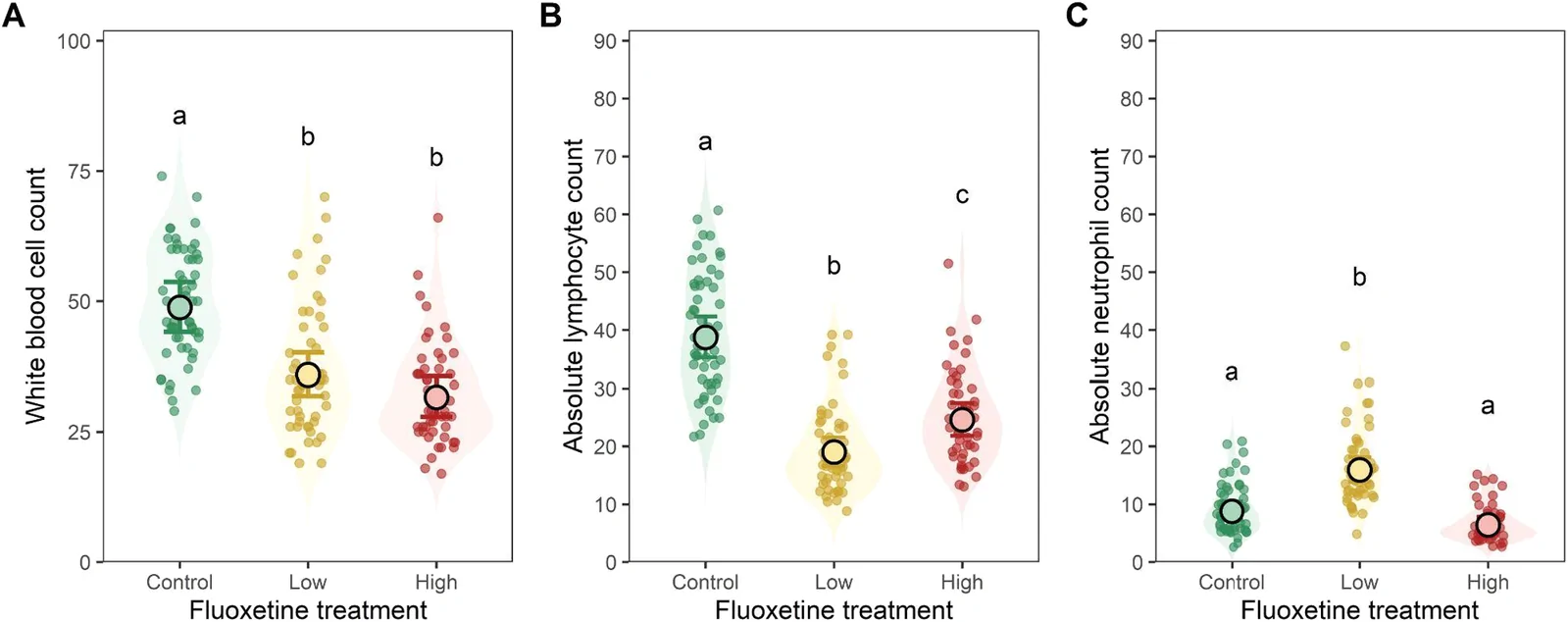
*Fluoxetine exposure altered baseline cellular immune traits in fish, reducing white blood cell and lymphocyte counts at both exposure levels while increasing neutrophil count under low fluoxetine exposure.* (A) White blood cell count, (B) absolute lymphocyte count, and (C) absolute neutrophil count across control, low, and high fluoxetine treatment. Violin plots represent the distribution of individual observations, colored points indicate individual fish, and large circles with error bars represent estimated marginal means ± 95% confidence intervals derived from generalized linear mixed-effects models (*N* = 160; Control = 54, Low = 53, High = 53). Different letters (a–c) indicate significant differences among treatments following Tukey-adjusted pairwise comparisons (*p* < 0.05).

Fluoxetine also had a significant impact on the absolute lymphocyte count (χ^2^ = 87.48, *df* = 2, *p* < 0.001) (Figure 4B). Both fluoxetine-exposed groups had lower lymphocyte counts than the control group (control vs low: estimate = 1.86 ± 0.20, *p* < 0.001; control vs high: estimate = 1.26 ± 0.20, *p* < 0.001). Lymphocyte count was also significantly lower in the low treatment group than the high treatment group (estimate = −0.60 ± 0.20, *p* = 0.011). Absolute neutrophil count varied significantly across different levels of fluoxetine (χ^2^ = 60.02, *df* = 2, *p* < 0.001) (Figure 4C). Fish exposed to low fluoxetine concentration had a higher neutrophil count than both the control (estimate = 1.03 ± 0.19, *p* < 0.001) and the high-exposure groups (estimate = 1.46 ± 0.19, *p* < 0.001). In contrast, neutrophil numbers in the high-exposure treatment were not significantly different from the control (estimate = 0.43 ± 0.19, *p* = 0.069).

Consequently, fluoxetine treatment resulted in significant changes in the neutrophil-to-lymphocyte (N/L) ratio (χ^2^ = 172.07, *df* = 2, *p* < 0.001). Fish exposed to low fluoxetine concentration had higher N/L ratios than both the control (estimate = 1.34 ± 0.11, *p* < 0.001) and the high groups (estimate = 1.18 ± 0.11, *p* < 0.001). In contrast, the high-exposure treatment did not differ significantly from control (estimate = 0.15 ± 0.11, *p* = 0.36).

## 4 Discussion

This study investigated the effects of fluoxetine exposure on behavioral responses, the skin mucus microbiome, and baseline cellular immune traits in rainbowfish. We found that these responses differed in their sensitivity to fluoxetine. Specifically, while neither behavioral traits nor microbiome diversity and composition were affected, fluoxetine significantly reduced the WBC count and altered the N/L ratio. Our results indicate that immune traits may represent a more sensitive target of fluoxetine exposure than behavioral or microbiome-associated responses. Therefore, integrating multiple biological systems is essential for understanding the ecological consequences of pharmaceutical pollution, as reliance on a single end point may underestimate biological impacts.

### 4.1 Complex Impact of Fluoxetine on Cellular Immune Traits

Fluoxetine exposure altered cellular immune traits in rainbowfish, with both exposure concentrations reducing total WBC count. This reduction suggests altered leukocyte dynamics, potentially through changes in cell production, survival, or redistribution to peripheral immune tissues involved in immune surveillance, such as skin, gills and mucosal surfaces.^15,37,65^ Furthermore, the WBC profile varied across exposure levels. Fish exposed to low fluoxetine concentrations showed a reduced lymphocyte count and an elevated neutrophil count, resulting in an increased N/L ratio. This finding is consistent with a stress-associated WBC redistribution response, whereby glucocorticoid-mediated immune modulation promotes lymphopenia (reduced lymphocyte numbers) and neutrophilia (increased neutrophil numbers), as previously reported in fluoxetine-exposed rainbow trout (*Oncorhynchus mykiss*).^15^ In contrast, fish exposed to high fluoxetine concentrations showed reduced lymphocyte counts, without a corresponding increase in neutrophils, suggesting that the regulation of different leukocyte populations may vary across fluoxetine concentrations rather than following a uniform stress-associated redistribution response.

Changes in circulating WBC numbers can have important ecological implications for host defense and disease susceptibility.^37,66,67^ The reduction in lymphocyte count observed in both treatments may compromise adaptive immune capacity by limiting antibody production and immunological memory.^68,69^ In contrast, the elevated neutrophil count observed only in fish exposed to the low fluoxetine concentration is consistent with a physiological stress response or inflammatory activation, reflecting the central role of neutrophils in rapid innate immune defense.^65,70^ These findings suggest that environmentally relevant fluoxetine exposure can alter circulating white blood cells associated with both adaptive and innate immunity, potentially influencing the capacity of fish to respond to pathogens and other environmental stressors. Future studies should therefore examine whether the effects of fluoxetine on cellular immune traits persist during a pathogen challenge.

The contrasting responses of individual leukocyte populations suggest that fluoxetine does not affect all measured cellular immune traits uniformly. Although both exposure concentrations reduced total WBC count, only the low concentration increased neutrophil count and, consequently, the N/L ratio, whereas lymphocyte count declined under both exposure levels. This divergence indicates that different leukocyte populations vary in their sensitivity to fluoxetine and may be regulated through distinct physiological mechanisms. Similar nonlinear dose–response patterns have been reported for other behavioral and physiological effects of fluoxetine in fish,^10,18,19^ suggesting that increasing exposure concentration does not necessarily result in proportionally greater biological effects. One possible explanation is that serotonergic and neuroendocrine pathways respond differently across concentration ranges through receptor desensitization, compensatory physiological feedback, or differential sensitivity among immune-regulatory mechanisms.^71–74^ While the precise mechanisms remain to be resolved, our findings demonstrate that cellular immune traits responded to environmentally relevant fluoxetine exposure despite the absence of detectable behavioral or microbiome responses, suggesting that immune function may represent a more sensitive indicator of pharmaceutical exposure than other commonly assessed biological end points.

### 4.2 Absence of Behavioral Effects

Despite significant effects on cellular immune traits, we found that fluoxetine exposure did not alter distance moved, freezing behavior, or shelter use in rainbowfish. This contrasts with studies reporting behavioral changes in other fish species exposed to SSRIs, particularly in traits related to boldness, exploration, and risk-taking.^10,18,19^ For example, fluoxetine exposure altered anxiety-related behavior in eastern mosquitofish (*Gambusia holbrooki*) after 28 days at 61 and 352 ng/L, with effects depending on sex and concentration, while sociability remained unaffected.^10^ Although exposure duration matched that of our experiment, the study assessed responses to different stimuli, in a different species and at different concentrations. These differences may help explain the contrasting behavioral findings. There is evidence that behavioral responses to fluoxetine can be context-dependent and vary by species, sex, life stage, exposure duration and concentration, and environmental conditions.^75^ For example, female mosquitofish exposed for the same 28-day period showed reduced responses to light–dark transitions at 18 ng/L, but not at 215 ng/L.^19^ Consistent with this, behavioral responses to SSRIs can often vary across studies even at comparable exposure concentrations.^10,19,76^ This variability likely arises because serotonergic disruption affects behavioral traits differently, while compensatory neural plasticity, individual coping styles, and variation in stress physiology can further modify behavioral responses among individuals and populations.^75–77^ The absence of behavioral effects despite significant changes in immune responses further supports the idea that behavioral and physiological end points can differ in their sensitivity to fluoxetine exposure.

### 4.3 Absence of Changes in Skin Mucus Microbiome

In fish, the microbiome associated with mucus is regulated by host mucosal immunity through mechanisms involving mucins, antimicrobial peptides, complement activity, lysozymes, and pattern-recognition pathways that collectively shape microbial colonisation and persistence.^78–81^ Consequently, physiological stressors that alter immune function, including SSRIs, are also expected to modify host-associated microbial communities, particularly when mucus integrity or inflammatory responses are disrupted.^25,82–84^ Because fluoxetine is known to affect immune regulation (see: Section 4.1), we anticipated that exposure to this antidepressant could alter the diversity and composition of the skin mucus microbiome. However, this was not the case, as no such effects were found.

Empirical evidence for SSRI effects on the skin mucus microbiome in fish remains limited, with most available studies focusing instead on gut-associated microbial communities.^32^ Here, fluoxetine-induced changes are typically dose-dependent and tend to emerge at relatively high concentrations, often exceeding environmentally relevant levels.^32^ Moreover, comparative evidence suggests that skin-associated microbial communities may be less responsive to moderate internal physiological changes than the gut microbiome, which is more directly influenced by host metabolism and dietary processes.^85^ Sampling time may also influence the detection of microbiome responses. Temporal changes have been reported in the gut microbiome of fluoxetine-treated mice^86^ and in fish skin microbiomes following environmental changes.^87^ Transient changes earlier in exposure followed by recovery therefore remain a possible explanation for the absence of detectable effects after 28 days. Sampling at multiple time points during exposure would help assess this possibility.

## Supplementary Material



## Data Availability

All raw data, processed data sets, R scripts, statistical analyses, figure-generation scripts, and knitted outputs associated with this study are publicly available in the GitHub repository: https://github.com/Melhado104/FLX_behaviour_immunity. The repository contains the complete reproducible workflow for behavioral, immune, and microbiome analyses conducted in this study.

## Notes

Summary statistics of fish body mass and standard length, summary statistics of measured fluoxetine concentrations in exposure water, temporal variation in measured fluoxetine concentrations throughout the exposure (PDF)

## ∇

U.A., A.P., and B.B.M.W. are senior authors
