# Supplementary material for: Psychoactive Pollutant Impacts Baseline Immune Traits but Not Behavior and Microbiome in a Fish
Source: Environ Sci Technol. 2026 Oct 20;60(43):30984–93. doi: 10.1021/acs.est.6c12057 (PMC13738779; doi:10.1021/acs.est.6c12057)
Supplement: Supplementary file 1 [file es6c12057_si_001.pdf]

Supplementary Materials

Psychoactive pollutant impacts baseline immune traits but not behaviour and microbiome in a fish

Authors and affiliations:

Gabriel Melhado[*,1], Rhiannon V. Eastment[1,2], Francine Z. Marques[3,4,5], Upama Aich[1,6,†], Anne Peters[1,†], and Bob B. M. Wong[1,†]

[1]School of Biological Sciences, Monash University, Melbourne, Victoria 3800, Australia

[2]School of Medical Sciences, Faculty of Biology, Medicine, and Health, University of Manchester, Manchester, Greater Manchester, M13 9PL, United Kingdom

[3]Department of Pharmacology, Biomedicine Discovery Institute, Faculty of Medicine, Nursing and Health Sciences, Monash University, Melbourne, Victoria 3800, Australia

[4]Victorian Heart Institute, Monash University, Clayton, Victoria 3168, Australia

[5]Baker Heart and Diabetes Institute, Melbourne, Victoria 3000, Australia

[6]Centre for Evolutionary Biology, School of Biological Sciences, The University of Western Australia, Perth, Western Australia 6009, Australia

[†] Senior authors

**Corresponding author:*

Gabriel Melhado - Email: gabriel.melhado@gmail.com

Table S1. Summary statistics of rainbowfish body weight (g) and standard length (cm).

Size metric	Treatment	Mean	SD	Min	Max
**Body mass**	Control	0.60	0.22	0.29	1.34
	Low	0.61	0.18	0.28	1.15
	High	0.62	0.21	0.33	1.26
**Body length**	Control	3.31	0.39	2.55	4.50
	Low	3.35	0.33	2.65	4.10
	High	3.34	0.36	2.78	4.21

Table S2. Summary statistics of measured fluoxetine concentrations (ng/L) in water samples collected throughout the exposure for the low- and high-fluoxetine treatments. Fluoxetine was not detected in control water samples (all measurements were below the analytical limit of quantification, LOQ = 2.0 ng/L).

Treatment	Mean	SD	*N*	Min	Max
Low	4.20	0.3	16	2.60	6.40
High	637.50	173.84	16	330.00	900.00

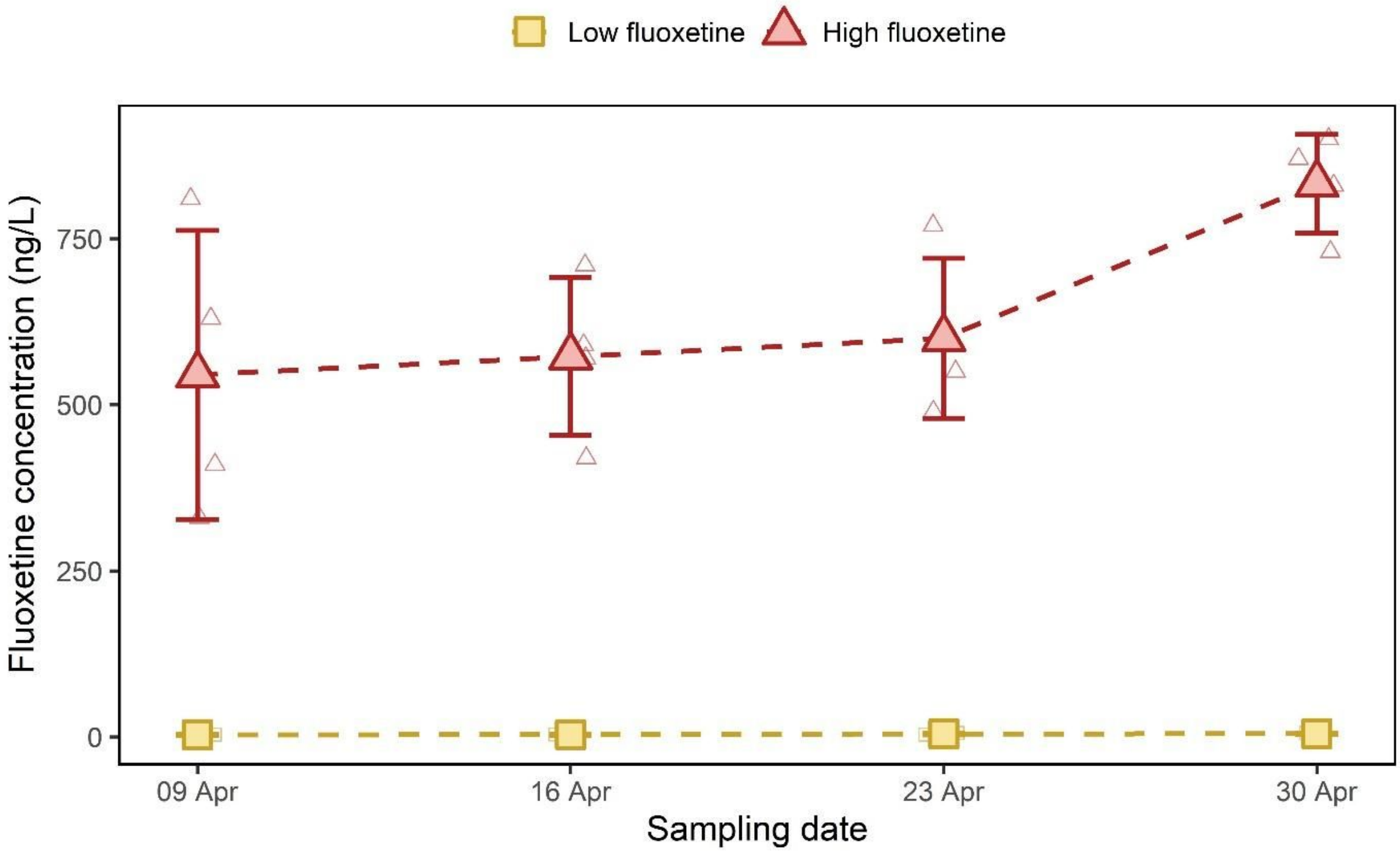

Figure S1. Measured fluoxetine concentrations (ng/L) in water samples collected from the low- and high-fluoxetine treatments throughout the exposure. Large symbols and error bars represent treatment means ± SD at each sampling date, whereas smaller symbols represent individual measurements from replicates.
